# Supplementary figures and images for: Single-cell RNA-seq analysis revealed the stemness of a specific cluster of B cells in acute lymphoblastic leukemia progression
Source: PeerJ. 2024 Oct 21;12:e18296. doi: 10.7717/peerj.18296 (PMC11505884; doi:10.7717/peerj.18296)

**A**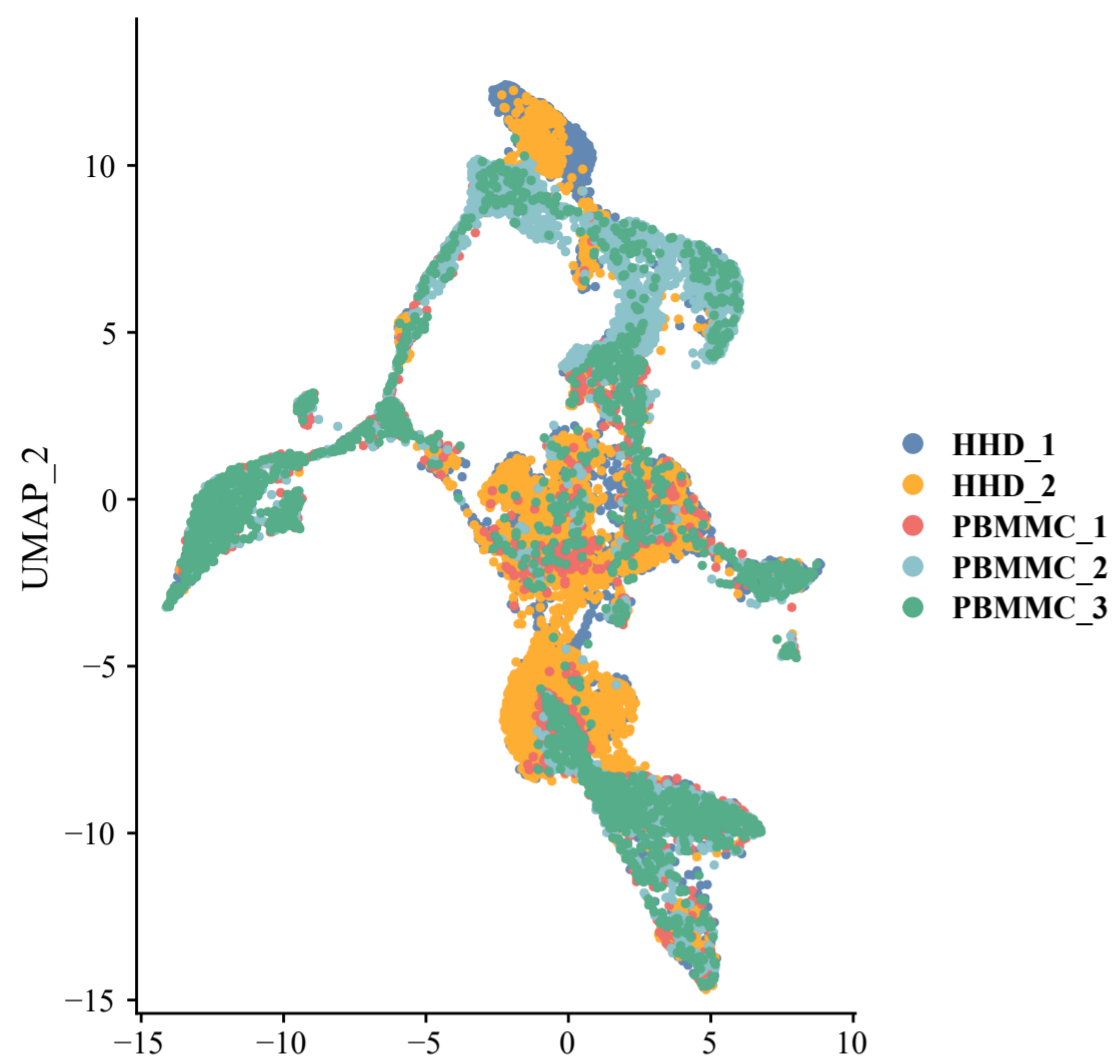**B**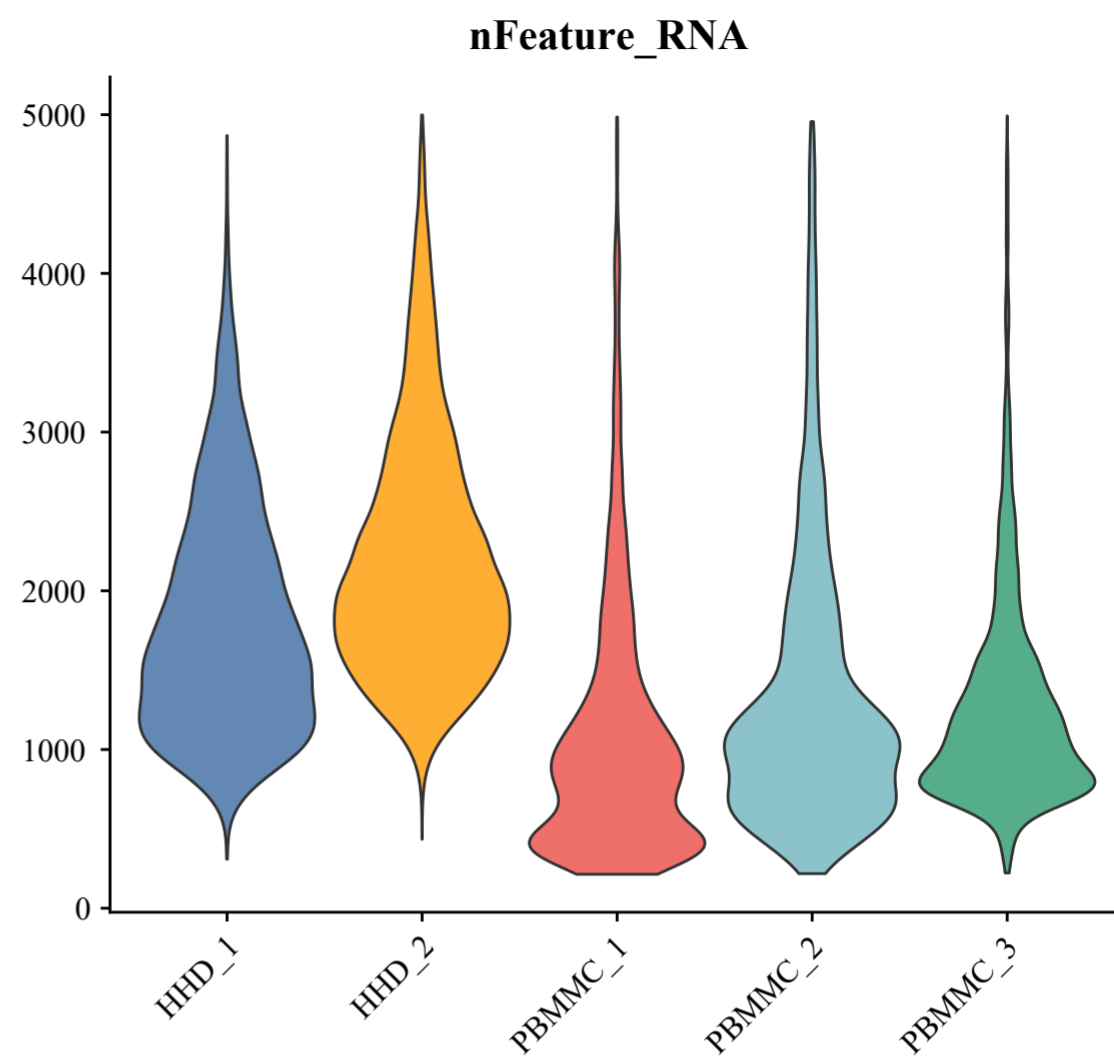**C**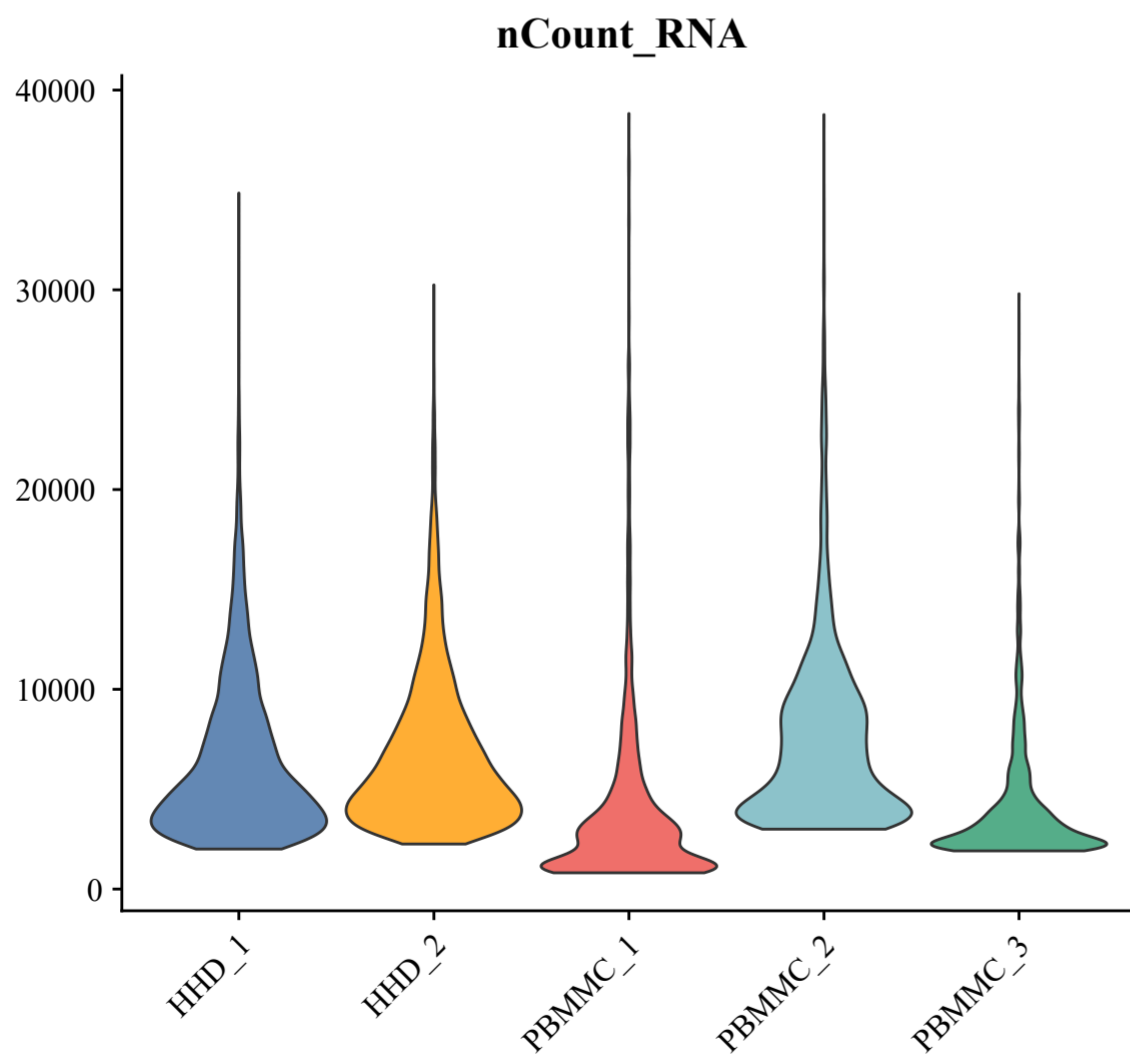**D**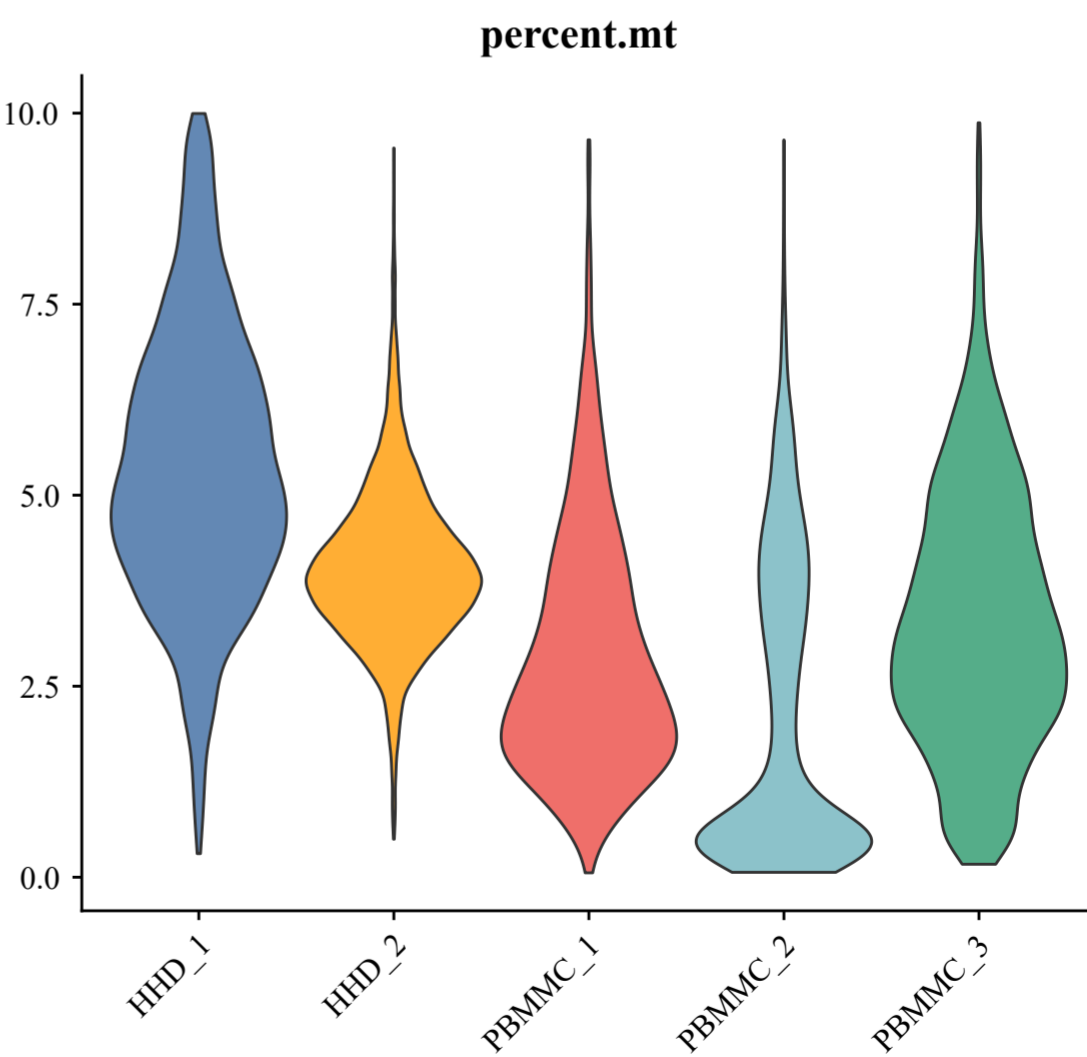

Supplement: Supplemental Information 1 — (A) UMAP of cell dimension reduction clustering in different samples. (B) The distribution of the number of genes in each cell. (C) Distribution of unique molecular identifiers (UMI) number in each cell. (D) The distribution of the proportion of mitochondrial genes in each cell. [file peerj-12-18296-s001.pdf]
